# Effect of Nitrogen Reactive Compounds on Aging in Seed

**DOI:** 10.3389/fpls.2020.01011

**Published:** 2020-07-08

**Authors:** Katarzyna Ciacka, Urszula Krasuska, Pawel Staszek, Agnieszka Wal, Joanna Zak, Agnieszka Gniazdowska

**Affiliations:** Department of Plant Physiology, Institute of Biology, Warsaw University of Life Sciences—SGGW, Warsaw, Poland

**Keywords:** germination, nitric oxide, seed viability, aging, vigor

## Abstract

Reactive nitrogen species (RNS) are universal compounds that are constantly present in plant cells. RNS function depends on their actual level (the “nitrosative door” concept), duration of plant exposure to RNS and the context of the exposure. RNS are involved in the nitration of nucleic acids and fatty acids, posttranslational protein modifications (nitration and *S*-nitrosylation), and modulation of reactive oxygen species metabolism. RNS are regulatory molecules of various physiological processes in plants, including seed formation, maturation, dormancy and germination. The free radical theory of aging, well documented for animals, indicated that RNS participate in the regulation of the life span. Some data point to RNS contribution in preservation of seed vigor and/or regulation of seed longevity. Seed aging is a problem for biologists and agriculture, which could be solved by application of RNS, as a factor that may potentially expand seed vitality resulting in increased germination rate. The review is focused on RNS, particularly nitric oxide contribution to regulation of seed aging.

## Introduction

Climate change causes weather extremes that influence plant mortality therefore, impacts biodiversity. Seed quality has an important bearing on the fate of the whole plant, and its development and lifespan. Seeds are the basis of plant production, the ultimate source of all food for humans and animals. The quality of seeds strongly influences the growth of the mature plants and determines their survival under environmental stress conditions.

The typical length of time that a seed survives (lifespan) varies among plant species. Seed longevity describes the length of time that seeds can remain viable and is an important factor for seedbanks in soil, seeds stored in warehouses, and the industries of seed production and sale (Walters et al., 2010; Sano et al., 2016). Moreover, seed lifespan is imprinted in the genes and influences efforts to preserve gene diversity in seedbanks (Walters et al., 2010).

**Figure 1:**
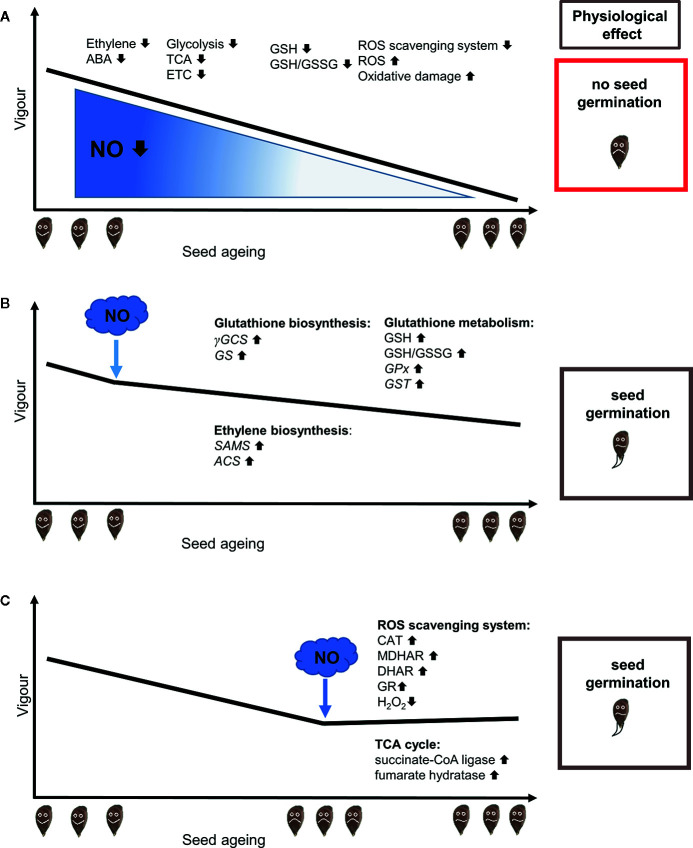
RNS application to maintain seed quality in aged seeds subjected to controlled deteriorated treatment (CDT) mitigates reduction of endogenous NO level. **(A)** A decrease in seed vigor during the aging process is induced by CDT. Seed aging is linked to ROS accumulation, due to impaired ROS generation and ROS scavenging by the antioxidant system, decreased GSH pool and GSH/GSSG ratio (Bailly et al., 2008; Ratajczak et al., 2019), disturbances in phytohormonal balance—decreased ethylene and ABA level (Sano et al., 2016) and down-regulation of the proteins involved in glycolysis, tricarboxylic acid (TCA) cycle, the electron transport chain (ETC) and oxidative phosphorylation (Xin et al., 2011). **(B)** The application of nitric oxide (NO) or NO donors before CDT activates protecting mechanisms that prevent the reduction of seed vigor. NO pre-treatment of the seeds subjected to CDT improves their quality by (i) increasing of GSH level and GSH/GSSG ration, (ii) up-regulation of the expression of genes encoding γ-glutamylcysteine synthetase (γGCS), glutathione synthetase (GS), glutathione *S*-transferase (GST), glutathione peroxidase (GPx), (iii) stimulation of methionine metabolism due to up-regulation of the transcription of the genes encoding *S*-adenosyl-L-methionine synthetase (SAMS) and 1-aminocyclopropane-1-carboxylic acid synthase (ACS) enzymes of ethylene biosynthetic pathway (He et al., 2018). **(C)** The application of NO or NO donors during CDT improves seed vigor by inducing processes that lead to the initiation of repair of oxidative damages, mainly in mitochondria. NO stimulates (i) activity of enzymatic antioxidant system in mitochondria: catalase (CAT), glutathione reductase (GR), monodehydroascorbate reductase (MDHAR), dehydroascorbate reductase (DHAR) (ii) leading to decreased H_2_O_2_ content in aged seeds, (iii) improves also mitochondrial function in aged seeds by enrichment of some proteins of TCA cycle: succinate-CoA ligase (ADP-forming) subunit and fumarate hydratase (Mao et al., 2018).

Seed longevity depends on internal and external factors (Sano et al., 2016). First of all, it is governed by the ability of seeds to withstand desiccation during maturation. *Recalcitrant*-type seeds are intolerant of water loss and are the most sensitive to aging, which is a major problem in their storage. *Orthodox*-type seeds are able to withstand low water content and are characterized by decreased metabolic activity. During long-term storage, seed longevity is determined by internal moisture content, external humidity, temperature, and oxygen pressure (Walters et al., 2010; Sano et al., 2016). Inappropriate storage conditions reduce seeds’ viability, and/or ability to germinate. Therefore, seed aging is associated with a reduction in longevity, mostly due to disturbances in metabolism and accumulation of harmful metabolites. Commonly, seed aging is associated with a loss of membrane integrity, modifications of nucleic acids, DNA degradation, impairment of protein and RNA synthesis, decreased energy metabolism (El-Maarouf-Bouteau et al., 2011 and citation therein; Corbineau, 2012). Furthermore, uncontrolled reactive oxygen species (ROS) generation, and inefficient antioxidant machinery are involved in the loss of seed vigor and viability (Bailly et al., 2008) (**Figure 1A**).

Seed aging is rather a slow process in the majority of plant species. Thus, artificial seed deterioration, including controlled deterioration treatment (CDT) or accelerated aging, are frequently applied in laboratory practice using established protocols (Bailly et al., 2008; El-Maarouf-Bouteau et al., 2011). Both treatments are based on application of sub-optimal conditions: elevated temperature and high humidity. The difference between CDT and accelerated aging is that during CDT seeds are imbibed to a precise water content prior to the warm temperature treatment (Corbineau, 2012). Such treatments are commonly performed to accelerate aging and produce uniformly aged seeds that are easy to examine and compare in scientific research. However, specific conditions of CDT cause some differences between artificially and naturally aged seeds. The present review addresses the role of reactive nitrogen species (RNS) in the regulation of aging in seeds, mostly in the context of CDT application.

## A Brief Introduction into Nitric Oxide (NO) Cellular Sources and Biosynthesis in Plants

For a long time, NO was recognized mainly as a pollutant of anthropogenic origin. Thereafter, it was discovered to be important in many physiological processes. NO was hailed as the “Molecule of the Year” by the journal *Science* in 1992. Experiments to discover NO biochemistry and its mode of action in living organisms were intensified after the 1998 Nobel Prize in Physiology and Medicine awarded for work on NO as a signaling molecule in the cardiovascular system. It was demonstrated that NO is emitted by plants, a discovery that launched 40 years of intensive research. The fine scheme of the milestone publications related to NO study in plants was done by Kolbert et al. (2019) and Del Castello et al. (2019).

NO, and compounds that are formed from the interaction of NO with oxygen or superoxide anion (O_2_^•−^) known as reactive nitrogen species (RNS), are generated in almost every cellular compartment (Durzan and Pedroso, 2002 and citation therein).

Biochemistry of NO is linked to the formation of its different redox forms: nitrosonium cation (NO^+^), nitric oxide (^•^NO), and nitroxyl anion (NO^−^). In aqueous solution O_2_^•−^, which belongs to ROS, rapidly reacts with NO and gives rise to peroxynitrite (ONOO^−^) and its protonated form—peroxinitrous acid (ONOOH) (Stamler et al., 1992 and citation therein; Durzan and Pedroso, 2002 and citation therein). NO also reacts with transition metal ions and other free radicals, and therefore it functions as an important regulator of metabolic processes (Stamler et al., 1992 and citation therein).

NO is synthesized *via* enzymatic and non-enzymatic pathways, which can be assigned to oxidative and reductive pathways. The best characterized and most acceptable enzymatic pathway of NO biosynthesis is a reaction catalyzed by nitrate reductase (NR) (Mohn et al., 2019). A recent report points on the primary function of the NIA1 isoform of NR as an NO-producing enzyme in Arabidopsis (Mohn et al., 2019). Moreover, some other molybdenum cofactor (Moco) containing enzymes are proposed to be involved in NO synthesis or homeostasis (Bender and Schwarz, 2018). An open question is whether higher plants possess an enzyme that is homologous to the mammalian NO synthase (NOS). The only known case of such an enzyme in the plant kingdom was found and extensively examined in a green alga, *Ostreococcus tauri* (Foresi et al., 2010). Although NOS-like activity (a reaction requiring all the cofactors and co-substrates of the mammalian NOS) has been measured in higher plants, no NOS homologues in genomes or transcriptomes of land plants were found in available databases (Kolbert et al., 2019). Doubts about the presence of L-arginine and the oxygen-dependent enzyme responsible for NO formation (like in mammalian NOS) led to the proposal that the term “NOS-like” be renamed “nitric oxide generating (NOG)” (Hancock and Neill, 2019).

The main non-enzymatic source of NO, and other reactive molecules derived from NO, is nitrite. Under acidic conditions, nitrite is protonated to nitrous acid (HNO_2_), which undergoes decomposition into different nitrogen oxides (NOx), depending on the redox state of the local environment of the reaction (Yamasaki, 2000; Rocha et al., 2011). The rate of NO release from nitrite is increased in the presence of a mild reductants (e.g., ascorbic acid) (Yamasaki, 2000).

In seeds, only a few pathways of NO generation are discussed. During germination, NO production is thought to start shortly after imbibition and to correlate with oxygen depletion (Bykova et al., 2015). Under low oxygen conditions, mitochondria are one of the main producers of NO in plant cells (Gupta et al., 2011); therefore, the mitochondrial electron transport chain could be a potential source of NO in seeds at early stages of germination. The non-enzymatic reduction of nitrite to NO has been demonstrated in the apoplast of the aleurone layers of germinating barley (*Hordeum vulgare* L.) caryopsis (Bethke, 2004).

The lowered oxygen concentration in germinating seeds promotes NO formation by NR. This pathway was confirmed in sorghum (*Sorghum bicolor* L.) and tomato (*Solanum lycopersicum* L.) seeds (Simontacchi et al., 2004; Lara et al., 2014).

Another putative mechanism of NO synthesis in oxygen-limiting conditions is a reaction catalyzed by xanthine oxidoreductase (Godber et al., 2000). During the first stages of germination, due to limited oxygen access, the oxidative routes of NO biosynthesis (hydroxylamine oxidation, NOS-like pathway) are unlikely. However, after the radical protrusion these pathways can be prevailing.

## NO—A Crucial Regulator of Seed Germination and Plant Senescence

NO plays a beneficial role as the universal regulatory molecule in plant physiology. In seed biology, its function is concentration-dependent and can be described by the model of the “nitrosative door” (Krasuska et al., 2015a), which is comparable to the concept of “oxidative window for germination” associated with critical range of ROS level (Bailly et al., 2008). Many previous studies have indicated the importance of NO in the regulation of seed dormancy and the transition from a dormant to non-dormant state (Šírová et al., 2011; Arc et al., 2013a; Bykova et al., 2015). The NO mode of action and its cross-talk with plant hormones in seeds have also been previously summarized (Arc et al., 2013b; Krasuska et al., 2015a; Sanz et al., 2015). Therefore, there is no doubt that NO is the modulator of seed germination, and the positive effect of this molecule on dormancy breakage has been observed. NO and its donors were used to break seed dormancy, or accelerate germination and improve the vigor of developing seedlings (He et al., 2014; Shams et al., 2018). Exogenous NO is also used in seed stratification treatment (Liu et al., 2019) to promote dormancy alleviation and enable faster germination.

At the molecular level, RNS are responsible for *S*-nitrosylation, tyrosine and tryptophan nitration of proteins, as well as nitration of fatty acids and nucleic acids (Corpas et al., 2015; Mata-Pérez et al., 2017; Arasimowicz-Jelonek and Floryszak-Wieczorek, 2019). Although such changes may constitute an integral signal transduction mechanism, in excess they cause degenerative processes linked to senescence, accelerated aging, or even cell death.

Evidence was provided that NO could be involved in the regulation of plant senescence. Treatment of soybean (*Glycine max* L.) cotyledons with NO deferred their aging *via* (among other effects) the stabilization of photosynthetic pigments (Jasid et al., 2009). In leaves, RNS contribute to the senescence process *via* interaction with ROS (Hung and Kao, 2003). Low level of NO caused by the expression of bacterial NO dioxygenase (NOD, the enzyme that converts NO into nitrate in the presence of oxygen) in Arabidopsis led to a senescence-like phenotype (Mishina et al., 2007). Fumigation of NOD-type plants with NO reduced the senescence phenotype, indicating that RNS delay senescence in leaves. There are no NODs in plants, nevertheless, the action of this enzyme is comparable to the hemoglobins’ (Hbs) (Gardner et al., 1998). Plants nonsymbiotic Hbs (nHbs) decrease NO concentration (Perazzolli et al., 2004; Igamberdiev et al., 2011). There is a high probability that dioxygenase reaction of the system involving nHbs may also regulate plant senescence.

## RNS in Seed Aging

An imbalance in cellular homeostasis and the time-dependent persistent alterations in the structure and function of biomolecules lead to the accumulation of cellular damages. These changes are universal features of aging in all living organisms, and they have been observed in aged seeds. Seed deterioration is a progressive, irreversible decrease in seed longevity accompanied by alterations of the nucleic acid structure (DNA fragmentation, chromosomal aberration, telomere length change, DNA methylation), lower capacity of the antioxidant system, and loss of membrane integrity. Deterioration process is also linked to the protein inactivation due to a variety of mechanisms, including non-enzymatic glycation through Amadori–Maillard reactions, oxidation of sulfhydryl groups, conversion of amino acids within the protein leading to partial folding or unfolding, dissociation to monomers or subunits, and condensation to polymers (El-Maarouf-Bouteau et al., 2011; Hu et al., 2012; Fu et al., 2015).

The loss of seed vigor is manifested in a reduced germination rate, reduced number of seedlings, and increased number of abnormal seedlings. In laboratory conditions acceleration of seed aging is obtained through application of different aging treatments e.g. CDT. CDT was used in studies focused on establishing the role of RNS in the maintenance of seed quality. He et al. (2018) investigated NO action in the regulation of aging in elm (*Ulmus pumila* L.) seeds induced by CDT (37°C and 100% relative humidity). CDT decreased the vigor of seeds to 50% after 2 days, but application of sodium nitroprusside (SNP) before CDT supported high vigor (**Figure 1B**). Furthermore, the treatment of elm seeds with SNP before CDT significantly increased their germination rate. A burst of NO was observed at the beginning of the CDT aging of seeds, and the endogenous NO content decreased as CDT progressed (He et al., 2018).

Similar data were reported for apple (*Malus domestica* Borkh.) embryos isolated from warm stratified seeds (subjected to accelerated aging). The maximum level of NO emissions occurred after 21 days of the treatment and was followed by the decline of NO emissions to the 70th day (Dębska et al., 2013). In general, apple seeds require a long period (3 months) of cold stratification for dormancy alleviation. Apple embryos that germinated after seeds were aged by warm stratification formed fewer seedlings, which also had developmental malformations (Dębska et al., 2013). It was previously demonstrated that developmental abnormalities can be reversed after NO fumigation (Krasuska et al., 2015b).

Oxidative damages due to the excessive formation of ROS are widely accepted to be the major contributors to seeds deterioration leading to their aging (Bailly et al., 2008; Kurek et al., 2019). Detoxification mechanisms, including antioxidant metabolites and enzymes responsible for modulation of the ROS content, limit oxidative damages to proteins, lipids, and nucleic acids. Therefore, NO plays a role in the regulation of aging in seeds by counteracting ROS generation and stimulating the antioxidant system (**Figures 1B, C**). ROS have many deleterious effects on mitochondrial membranes, resulting in the release of cytochrome *c* into the cytosol to activate apoptotic cell death during the loss of seed viability (reviewed by Fu et al., 2015). Mitochondrial DNA is susceptible to ROS-induced damages, which lead to dysfunction of the organelles and are considered to be a major component of seed aging.

Mao et al. (2018) described the accumulation of H_2_O_2_ in mitochondria in artificially aged oat (*Avena sativa* L.) seeds that exhibited low vigor. The application of SNP to aged oat seeds had a protective effect, improving seed vigor and increasing ROS scavenging ability in mitochondria (**Figure 1C**). Higher activities of catalase, glutathione reductase, monodehydroascorbate reductase, and dehydroascorbate reductase in the ascorbate–glutathione (AsA–GSH) antioxidant system were also noticed. The activity of mitochondrial complex IV in the aged oat seeds decreased, but after application of NO, the activity increased to the level found in a non-aged caryopsis (Mao et al., 2018). This was a striking finding, because NO is considered an inhibitor of cytochrome *c* oxidase. Mao et al. (2018) suggested that alternative oxidase was stimulated by NO, which may partly explain the decline in ROS production. In addition, protein abundance levels of some tricarboxylic acid cycle (TCA) enzymes, succinyl-CoA ligase and fumarate hydratase, in mitochondria from aged oat seeds increased after SNP treatment (**Figure 1C**). Thus, it was proposed that the NO treatment of aged seeds, could increase the capacity of some reactions of the TCA cycle and also the AsA–GSH cycle, leading to a lowering of the mitochondrial H_2_O_2_ content (Mao et al., 2018). GSH is a marker of seed vigor, and a low GSH/GSSG ratio is linked to seed aging and loss of vigor (Kranner et al., 2006) (**Figure 1A**). SNP pre-treatment of elm seeds subjected to CDT induced the expression of genes that encoded enzymes of the glutathione biosynthetic pathway and led to an increased level of GSH, which can protect seeds from oxidative damages resulting from ROS over-accumulation (He et al., 2018).

The vigor of aged seeds depends on ethylene emission and seeds’ sensitivity to this hormone (Siriwitayawan et al., 2003). The physiological state and germination potential of seeds are linked to ethylene-NO cross-talk, ethylene biosynthesis, and signaling (Arc et al., 2013b). The interaction of NO and ethylene during dormancy release in apple embryos was demonstrated (Gniazdowska et al., 2010). Embryos sensitivity to this hormone (regarded as a beneficial germination factor) increased after short-time fumigation with NO. In the context of seed aging, it was shown that pre-treatment of elm seeds (before CDT) with NO donors (SNP, GSNO) prevented a drastic decrease of expression in genes encoding ethylene biosynthesis enzymes: *S*-adenosyl-L-methionine (SAM) synthetase and 1-aminocyclopropane-1-carboxylic acid (ACC) synthase (ACS) (He et al., 2018) (**Figure 1B**). Taking into account that, on one hand, ethylene promoted germination of Arabidopsis seeds subjected to salinity stress (which induced oxidative stress), but on the other hand, SNP upregulated *ACS2* expression, resulting in a lower H_2_O_2_ level (Lin et al., 2013), it appears that the NO-ethylene synergistic interaction delays seed aging caused by oxidative stress.

In contrast, high ethylene emission typically occurs during senescence of leaves or petals and during fruit ripening (Li et al., 2013). ACC in the presence of NO can be converted *via* non-enzymatic reaction to ethylene (Gniazdowska et al., 2010). This raises the question whether an over-accumulation of RNS and ROS could stimulate non-enzymatic ethylene emission during seed aging (even during dormancy), which may accelerate deterioration.

SAM is the metabolite that links biosynthesis of NO, ethylene, and polyamines (PAs) (Krasuska et al., 2014; Krasuska et al., 2016). PAs are regulators of plant growth and development that also modulate seed aging. Their content generally decreases during seed aging (Matilla, 1996), and they are known to maintain vigor and viability during accelerated aging (Yalamalle et al., 2019). A reduction in the PAs level seems to be a significant prelude to senescence signals (Chen et al., 2019). In contrast, germination of apple embryos induced by NO fumigation was associated with increased activity of polyamine oxidase (PAO) (Krasuska et al., 2014). Conversion of spermine by PAO results in liberation of H_2_O_2_, the key molecule for seed dormancy breakage. Thus, NO-stimulated catabolism of PAs in seed aging is unwelcome, while NO-dependent H_2_O_2_ formation due to an enhancement of PAO activity may be beneficial during seed germination. However, prolonged NO action could be potentially destructive because it stimulates the generation of harmful ROS. So again, whether NO plays a positive or negative role in seed biology, including acceleration of aging, is concentration- and time-dependent.

Seed germination, vigor, and aging were linked to ABA control (Arc et al., 2013b). In dormant apple embryos, NO fumigation reduced sensitivity to ABA (Gniazdowska et al., 2007) as well as expression of the transcription factor abscisic acid insensitive 5 (*ABI5*) (Andryka-Dudek et al., 2019). Schausberger et al. (2019) used seeds of two lines of Chinese kale (*Brassica oleracea* L.) with different ABA content to show that a low endogenous ABA level increased sensitivity of seeds to artificial aging. In ABA-deficient or insensitive Arabidopsis mutants, reduction of seed longevity was clearly associated with a lack of dormancy (Sano et al., 2016).

NO participates in post-translational protein modifications (PTMs), including *S*-nitrosylation of proteins, which was suggested to play a beneficial role in seed germination. In Arabidopsis seeds, *S*-nitrosylation of ABI5 promoted germination by stimulating ABI5 protein degradation (Albertos et al., 2015). Furthermore, markedly decreased levels of SNO-proteins during prolonged seed aging can be recognized as a signal for selective protein degradation, similar to that observed for ABI5 (Albertos et al., 2015). Protein *S*-nitrosylation is a reversible PTM that was shown to enhance the activities of antioxidant enzymes, thereby reducing cellular ROS levels. Therefore, *S*-nitrosylation can indirectly protect against uncontrolled protein carbonylation (an irreversible PTM occurring as a reaction to oxidative stress) (Bai et al., 2011). It was demonstrated in recalcitrant *Antiaris toxicaria* Lesch. seeds, that pre-treatment with NO stimulated germination by causing *S*-nitrosylation of antioxidant enzymes, which modified their activities (Bai et al., 2011).

In elm seeds, *S*-nitrosylated proteins were accumulated at an early stage of aging (He et al., 2018). The study of He et al. (2018) showed that *S*-nitrosylation in elm seeds affected proteins of carbohydrate metabolism that participate in glycolysis, the mitochondrial TCA cycle, and pentose phosphate pathways. The authors concluded that *S*-nitrosylation, and therefore NO signaling, can be one of the mechanisms involved in regulation of deterioration process in seeds.

## Conclusions

Aging leads to a decrease in the quality of seeds (**Figure 1A**), which limits not only agricultural production but also the preservation of global biodiversity. Studies aiming to understand the mechanisms underlying seed aging and the associated decrease in seed quality employ the artificial accelerated aging approaches that allow to obtain a pool of equally aged seeds. Treatments that effectively protect seeds against aging or prevent seed deterioration require further evaluation.

NO and other compounds belonging to the RNS family appear to mitigate the negative effects of seed aging. Treatment of seeds with NO or NO donors before induction of aging, or at the initial stages of aging (**Figures 1B, C**), activates the antioxidant system, which delays or prevents the initiation of mechanisms that induce aging. The application of NO donors at some stages of the aging process activates defence mechanisms (e.g., reversible redox PTMs, AsA–GSH cycle), which lead to an improvement in seed quality (**Figure 1C**), even if the aging process was activated earlier. Although evidence has been provided that NO can partially prevent seed deterioration caused by aging, its application in the seed industry requires further research.

## Author Contributions

Conceptualization: UK, KC, PS, AG. Writing—original draft preparation: UK, KC, AG. Writing—editing and figure preparation: PS, JZ, AW. Supervision: AG. Funding acquisition: UK. All authors contributed to the article and approved the submitted version.

## Funding

This work was performed during realization of the project financed by National Science Centre, Poland 2016/23/B/NZ9/03462 given to UK.

## Conflict of Interest

The authors declare that the research was conducted in the absence of any commercial or financial relationships that could be construed as a potential conflict of interest.
